# Treatment of Intermittent Fever

**Published:** 1846-12

**Authors:** 


### EDITORIAL DEPARTMENT.

*Treatment of Intermittent Fever.*—We have noticed in our exchanges of late, several articles by practical physicians communicating their experience in the treatment of intermittents by an immediate resort to the specific remedy, without premising cathartics, emetics, &c., which are recommended by all systematic writers, and generally prescribed by the profession. We are gratified to see accumulating testimony of the superiority of this plan of treatment. We became convinced, from our own experience, of its propriety and advantages several years ago, and communicated our views in an article published in the American Journal of Medical Sciences, October, 1841. Since that period we have always pursued this course, except in some instances in which we have felt compelled to yield in some degree to the determined prejudices of patients, derived from general usage, and the popular (as well as professional) idea that a course of evacuations of the chylopoetic viscera is requisite, as a prelimnary measure, in all cases.

In treating intermittents, if we can exercise fully our judgment and choice, we never delay the administration of quinia, unless at our first visit we find the patient in the paroxysm. Under these circumstances we employ measures to render the paroxysm as complete as possible, directing warm diluents, and prescribing generally a few grains of Dover's powder. Directly the sweating stage is concluded, nor, if it be very desirable to gain time, do we wait even for that, we prescribe four or five grains of the quinia in pill, powder, or solution, as the patient prefers. We direct this dose to be repeated morning, noon and night. We can recollect but few instances in which this quantity has not been well borne, unless in cases of gastric irritability, which occasionally, but not very often, is a source of embarrassment. If the type be quotidian, the patient will be almost sure to have another paroxysm. If it be of the tertian type, the chances are about even that he will escape the next. The chances are three to one, in the case of the quotidian, that a third paroxysm will not occur, and the liabilities to a fourth paroxysm are at Zero. In the case of the tertian, the chances of a third paroxysm are nearly nothing. These computations are estimated, not based upon an actual enumeration of cases; yet, we have no doubt that any practitioner, treating the disease in a locality like this, will find them verified by pursuing the same practice. Instead of proceeding at first to give emetics and cathartics, we invariably reject the former, and endeavor, if possible, to avoid the latter. Even if the patient be costive, we usually defer moving the bowels for a day or two, except by an enema. And then we direct the mildest means, and endeavor so to time the operation of the laxative that it shall not be likely to occur at, or near the period when the paroxysm is expected. We believe purging at that time, and at any time with this disease, to be not only unnecessary, but positively disadvantageous. It is very apt to occasion a recurrence of the paroxysms when they would otherwise have been arrested. Calomel, to "improve the secretions," "excite a flow of bile," and other suppositious ends, we have entirely discarded. The blue pill, however, as a laxative is one of the best that can be selected. Our principle, in short, is to cure the disease as soon as practicable by a prompt resort to the specific remedy in adequate doses, believing, as we do, that the gastric disorder, &c., are *effects* rather than *causes* of the disease, and will be increased rather than diminished by delay. The remedies employed (cathartics and emetics) to relieve the disorders accompanying the disease, so far from doing this, in the majority of cases, tend to aggravate them, or to produce them if they do not already exist. We would pursue the same course whatever complications existed: active inflammation of a vital viscus, would, in our view, constitute no objection. The doses of quinia which we employ, a few years ago would have been deemed *large doses;* now they are but medium, but they are adequate to the end to be attained, and, surely, nothing beyond this is desired. We do not wish, in treating this affection, to give as much quinia as the patient can bear with safety, but to give enough to cure the disease *cito, tuta et Jucunde;* and for this it is rarely necessary to exceed the quantity stated.

We deem it important that the patient continue the remedy three or four weeks after the paroxysms are interupted, but in diminished doses. Our general rule is to direct about eight grs. daily for the first week, four grs. for the second week, and two grs. for the third and fourth weeks. If the patient be feeble and anæmic, the latter portion of the time it may be advantageously combined with some of the salts of iron, and under these circumstances it is frequently useful to continue the combination much longer.

We permit our patients to take animal food as soon as their appetite returns, and frequently conjoin with a nutritious diet a little wine. Patients having once had this disease will almost invariably suffer from relapses. Contrary to a doctrine inculcated by some practical writers, and, also, to an impression which is quite popular, the liability to relapses is inversely in proportion to the length of time the disease has been permitted to go unchecked. The patient should always be provided with the remedy, and as soon as any intimations of its approach are noticed, he should resort to it at once. In this way many relapses are warded off. A symptom which we have frequently found to precede an attack, is turbidity of the urine from an excess of the lithate of ammonia. We are therefore in the habit of advising patients to watch the appearance of this secretion, and whenever it is high colored, and deposits on cooling the lateritious sediment, to prepare for a relapse — or rather, to provide *against* it.

Managed in this way intermittent fever, in this locality at least, is to be regarded as comparatively a trivial disease. To those of our brethren who do not coincide with us in the propriety of the mode of practice which we have sketched, we have only one word farther to say—*give it a fair trial, and then decide upon its merits.*

